# Fiscal Incentives and Health Risk Protection: How Central–Local Fiscal Relations Shape Rural Households’ Medical Burden in China

**DOI:** 10.3390/healthcare14050649

**Published:** 2026-03-04

**Authors:** Yitong Zhang, Pengju Liu, Tao Li, Lingdi Zhao

**Affiliations:** 1 Department of Economics, Ocean University of China, Qingdao 266100, China; zhangyitong1211@stu.ouc.edu.cn (Y.Z.); lingdizhao512@ouc.edu.cn (L.Z.); 2 Department of Computer Science & Technology, Qingdao University of China, Qingdao 266071, China; liupengju@qdu.edu.cn; 3 Department of Economics, Qingdao University of China, Qingdao 266071, China

**Keywords:** local fiscal matching subsidies, medical economic risks, central transfer payments, moderating effect, mediating effect

## Abstract

**Highlights:**

**What are the main findings?**

- Local fiscal matching subsidies significantly reduce catastrophic health expenditures for rural households under the income effect of central transfer payments but may increase them under substitution effects due to non-cooperative behavior by local governments.
- New Rural Cooperative Medical System (NCMS) compensation spending serves as a mediating channel, and the effects exhibit significant regional heterogeneity, being more pronounced in eastern regions, areas with a more developed secondary industry, and regions with higher fiscal self-sufficiency rates.

**What are the implications of the main findings?**

- The findings provide new evidence on how intergovernmental fiscal incentives shape medical economic risks under the NCMS.
- It offers actionable policy implications for fiscal and healthcare reforms in countries with similar contexts to China, particularly regarding defining local fiscal matching boundaries and leveraging transfer payments to advance the integration of urban-rural basic medical insurance.

**Abstract:**

**Background**: The majority of the funding for the New Rural Cooperative Medical System (NCMS) is derived from fiscal subsidies, comprising central transfer payments and local fiscal matching subsidies. Local governments’ strategic behavior in response to central transfer payments may further impact NCMS compensation spending and medical economic risks. **Methodology**: Accordingly, this paper investigates, from both theoretical and empirical perspectives, the impact pathways through which local fiscal matching subsidies influence the medical economic risks faced by insured rural households, with central transfer payments serving as a moderating factor. This paper constructs a dynamic game framework involving the central government, local governments, and household sectors. It further applies a mediation effect model and related econometric methods to conduct empirical analysis using 87,630 observations from the China Family Panel Studies (CFPS). **Results**: The results show that, first, local fiscal matching subsidies significantly reduce catastrophic health expenditures for rural households under the income effect of central transfer payments. However, under the substitution effect, the opposite occurs, as local governments adopt non-cooperative strategies in response to central transfer payments. Second, these impacts exhibit regional heterogeneity, with stronger effects in eastern regions, regions with more developed secondary industries, and regions with higher fiscal self-sufficiency rates. Third, local fiscal matching subsidies influence medical economic risks through compensation spending, under the moderating role of central transfer payments. **Conclusions**: This paper provides a novel perspective on why the NCMS struggles to provide effective protection, thereby enriching the existing literature. Furthermore, it provides policy guidance for fiscal and healthcare reforms in countries with similar contexts to China. Based on these insights, we argue that, during the future integration process of the Basic Medical Insurance for Urban and Rural Residents, clear boundaries should be defined for local fiscal matching subsidies, and the moderating role of central transfer payments should be strategically leveraged.

## 1. Introduction

The World Health Organization (WHO) emphasizes that high medical expenses directly affect household economic behavior and may push families to the brink of financial distress. Although the overall rural poverty rate has decreased from 10.2% in 2012 to 0.6% in 2019, illness-induced poverty remains the primary factor leading rural households into poverty. To mitigate medical economic risks, the Chinese government has established a multi-tiered healthcare security system centered around basic medical insurance. For rural residents, the New Rural Cooperative Medical System (NCMS) serves as the primary institutional pathway for accessing healthcare protection. However, despite rapid economic growth, persistent disparities between urban and rural development have persisted. Compared with urban residents, rural residents have lower and more unstable income levels, which reduces their ability to afford medical expenses. In addition, with the current acceleration of population aging and rising incidence of chronic diseases, healthcare needs have expanded from “basic diagnosis and treatment” to “long-term care” and “major illnesses treatment”. Against this backdrop, issues such as the limited protection level of the NCMS, weak rural medical services, and inadequate institutional fairness have gradually emerged. In response to these challenges, the basic medical insurance system is entering a new phase of integrated urban-rural development. During this period, various regions have continuously raised the NCMS funding levels for rural insured populations. Regarding the financing structure, fiscal subsidies are the primary source of NCMS funding, while individual contributions constitute a relatively small proportion. Theoretical research suggests that fiscal subsidies reduce insurance premiums for rural households, increase their willingness to participate, ensure adequate compensation for sick rural households under the NCMS, and enhance their capacity to withstand medical economic risks [1]. However, in practice, the financing relationship between the central and local governments is far more complex. The tax-sharing system encourages the central government to delegate responsibility for NCMS matching subsidies to local governments while simultaneously balancing regional financing disparities through central transfer payments based on each region’s level of economic development [2]. Therefore, to better understand the mechanisms by which local fiscal subsidies affect medical economic risks, it is essential to consider the relationship between central transfer payments and local fiscal matching subsidies embedded within NCMS funding, as well as the resulting compensation behavior of local governments.

Existing studies have not provided a consistent answer regarding whether the NCMS can reduce catastrophic health expenditure. Given the government’s ongoing increase in fiscal subsidies, attributing the system’s failure at providing adequate protection solely to insufficient compensation spending is unconvincing. Furthermore, the current literature has primarily examined the fiscal risks associated with the NCMS and its urban-rural integration from two perspectives: the sustainability of fiscal subsidies [3,4,5,6,7,8,9], and the equity implications of such subsidies [10,11,12,13]. However, few studies have directly examined how the NCMS affects medical economic risks from the perspective of fiscal relations between central and local governments. This leaves a research gap in understanding intergovernmental strategic interactions within the NCMS risk-sharing mechanism. Following the research framework of Fan and Wang [14], this paper argues that the dual incentive of economic decentralization and promotion mechanisms causes central transfer payments to generate both income and substitution effects on local fiscal matching subsidies. The income effect increases NCMS compensation spending, thereby raising expected medical expenditure and reducing medical economic risks (Hypothesis 1). The substitution effect on compensation spending is jointly determined by the fiscal revenue effect and the crowding-out effect: the fiscal revenue effect increases compensation spending, raises expected medical expenditure, and reduces medical economic risks, whereas the crowding-out effect on productive expenditures produces the opposite impact (Hypothesis 2). Accordingly, this paper verifies and analyzes the two hypotheses from both theoretical and empirical perspectives.

Given the limited attention to how intergovernmental fiscal incentives affect the healthcare protection of medical insurance, and the practical demand for optimizing the financing mechanism in the integration of urban and rural basic medical insurance, this paper makes two marginal contributions: First, it investigates the mechanism through which local fiscal matching subsidies affect medical economic risks from a novel perspective, and systematically analyzes how these subsidies are shaped by the moderating effects of central transfer payments and subsequently alter rural households’ medical spending behavior via NCMS compensation spending, thereby affecting their medical economic risks. Second, using microdata from the China Family Panel Studies (CFPS) and combining it with macro-level data on central transfer payments and local fiscal matching subsidies, this paper empirically tests the impact of local fiscal matching subsidies behavior on medical economic risks under the moderating effect of central transfer payments. The findings indicate that inefficiencies in local fiscal matching subsidies are the fundamental cause behind the limited effectiveness of NCMS compensation in reducing catastrophic health expenditure among insured rural households. Overall, this paper offers a novel perspective on why the NCMS struggles to deliver effective protection, thereby enriching the existing literature. In addition, the findings provide analytical references for fiscal and healthcare reforms in other countries with institutional contexts similar to that of China.

This paper consists of eight sections. The Section 1 is the introduction, i.e., the research background. The Section 2 outlines the institutional background relevant to this paper. The Section 3 reviews the literature and develops theoretical hypotheses. The Section 4 highlights the data and methodologies employed in the empirical analysis. The Section 5 analyzes the empirical results, including basis regression, endogeneity discussions, robustness tests, heterogeneity tests, and mechanism tests. The Section 6 presents a discussion that provides an in-depth examination of the findings and their practical implications. The Section 7 and Section 8 summarize the conclusions and policy suggestions.

## 2. Institutional Background

The tax-sharing system, introduced in 1994, marked the beginning of China’s fiscal decentralization reform. The external objective of this system was to raise two key ratios: the share of central government revenue in total fiscal revenue and GDP. The underlying reform strategy, however, involved the central government delegating some fiscal authority to local governments while assigning them responsibility for public services and using transfer payments to balance service disparities across regions. Throughout the development of the NCMS financing, shifting responsibilities between the central and local governments can be clearly traced as the tax-sharing reform progressed.

In 2003, to mitigate the difficulties and high costs associated with medical care for rural residents and reduce the financial burden on rural households, the State Council issued the Opinions on Establishing a New Rural Cooperative Medical System. To ensure equitable financing, the NCMS adopted a funding principle that combined individual contributions, government fiscal subsidies, and collective support. However, in practice, the government assumed the primary responsibility for financing. Specifically, this funding relationship follows a model characterized by “central government leadership, local government co-responsibility, and regional redistribution”. Under this model, the central government takes primary responsibility through high-ratio subsidies, while local governments fulfill their matching funding obligations through a tiered system at the provincial, municipal, and county levels. Since 2006, the central government has provided modest subsidies to insured populations in eastern regions, while implementing a 1:1 matching arrangement with local governments for central and western regions. During the transition from pilot to full coverage (2003–2010), the proportion of local subsidies remained above 60% [15]. As the NCMS was fully rolled out (2010–2017), the standard for fiscal subsidies increased annually by RMB 40. Regarding the incremental portion of these subsidies, the central government continued to provide subsidies at an appropriate ratio for insured populations in eastern regions. However, it substantially increased support for central and western regions, with the central government’s share reaching 60% and 80%, respectively [16]. During this phase, the proportion of central government subsidies gradually exceeded that of local subsidies. In July 2018, the State Council issued the Notice on Reforming the Division of Fiscal Powers and Expenditure Responsibilities between the Central and Local Governments in the Health Sector, which categorized 29 provincial-level administrative units and five separately administered cities into five tiers, further detailing the central government’s contribution ratios for additional subsidies. Among these, ten central provinces, including Hebei, and twelve western provinces, including Inner Mongolia, were classified as Tier 2 and Tier 1, respectively, with subsidy ratios remaining at 60% and 80%. Liaoning, Fujian, and Shandong were classified as Tier 3, receiving subsidies at a rate of 50%. Tianjin, Jiangsu, Zhejiang, Guangdong, and the five separately administered cities were placed in Tier 4, with a subsidy rate of 30%. Beijing and Shanghai were designated as Tier 5, with a subsidy rate of 10%.

It is evident that a key feature of local fiscal subsidies is the provision of varying transfer payment standards by the central government to various regions according to their economic development status. Typically, central-to-local transfer payments can be classified into two categories: general transfer payments and special-purpose transfer payments. General transfer payments, also known as unconditional transfer payments, primarily aim to balance fiscal budget constraints arising from differences in resource endowments across regions, thereby maintaining relative symmetry between fiscal authority and responsibilities at the local level. In contrast, special-purpose transfer payments are intended to achieve specific macroeconomic and social objectives. Some of these special-purpose transfer payments require matching funds from local governments; hence, they are also termed conditional transfer payments. At the inception of the NCMS, to facilitate its nationwide implementation, the central government adopted a special-purpose funding approach for local NCMS transfer payments. After 2011, to enhance the equalization of public services across regions, the central government reclassified NCMS transfer payments as general transfer payments [17,18]. Specifically, to align with the division of fiscal powers and expenditure responsibilities between the central and local governments in the health sector, the Ministry of Finance, in conjunction with the National Healthcare Security Administration, issued the Management Measures for Central Financial Subsidies for Urban and Rural Residents’ Basic Medical Insurance in September 2019. This notice explicitly categorized central financial subsidies for urban and rural residents’ medical insurance into a newly established type of general transfer payments, termed “joint fiscal powers transfer payments”. Nevertheless, the annual incremental portion of NCMS fiscal subsidies continues to require corresponding matching funds from local governments. Therefore, it is evident that the current NCMS transfer payments continue to demonstrate path dependency toward special-purpose transfer payments, and their moderating effect on local fiscal matching subsidies cannot be overlooked.

## 3. Literature Review and Theoretical Hypothesis

### 3.1. Literature Review

#### 3.1.1. NCMS and Medical Economic Risks

To reduce the financial burden of illness on rural residents, China established the NCMS in 2003, reaching near-universal coverage by 2010. Scholars have debated the relationship between the NCMS and medical economic risks. Theoretically, medical insurance mitigates the risk of catastrophic health expenditure by reducing out-of-pocket expenses and lowering the cost of healthcare services through compensation mechanisms [19,20,21]. However, empirical evidence on the impact of the NCMS on medical economic risks remains inconclusive. Some studies have found that the NCMS provides a degree of risk compensation, alleviating the financial pressure and medical expense burden borne by impoverished patients. By reducing and eliminating future uncertainties, it can further alleviate poverty conditions [22,23,24,25,26,27,28]. However, some studies indicate that, although issues such as low enrollment rates and inconvenient reimbursement procedures have gradually been addressed as the NCMS has developed, the financial burden on rural households persists [29]. This is because the reimbursement rate under the NCMS averages approximately 60%, which is lower than that of urban resident medical insurance and urban employee medical insurance. The relatively inadequate compensation level implies that the effect of the NCMS in alleviating the financial burden of medical expenses for rural households is substantially lower than for urban residents [30,31,32]. It struggles to meet multi-tiered healthcare needs, resulting in an increase rather than a decrease in their medical economic risks [33,34,35]. According to the China Health Statistics Yearbook, NCMS per capita funding in China continued to increase, while average medical expenses per hospitalized patient also rose steadily. Consequently, medical economic risks remain a persistent challenge. These findings indicate that attributing the insufficient risk mitigation effect of the NCMS on rural household medical financial burdens solely to inadequate compensation is unpersuasive. Hence, scholars should explore additional factors contributing to this issue.

#### 3.1.2. The Central–Local Relationship in NCMS Medical Economic Risks Sharing

The 1994 tax-sharing reform strengthened the central government’s fiscal coordination and macro-governance capacities by centralizing fiscal authority while decentralizing expenditure responsibilities. However, it also led to a significant shortage of local autonomous fiscal capacity, thereby constraining regional economic development [36]. In response, the central government established a fiscal transfer payments system to address local fiscal gaps and foster regional economic development. For local governments, central transfer payments represent a common-pool resource. While local governments derive the majority of benefits from utilizing these funds to enhance public services and stimulate economic development, they incur only a fraction of the associated costs [37]. This incentivizes local governments to increase fiscal expenditures while reducing their own fiscal revenues, thereby widening local fiscal gaps to capture more of these “low-cost” transfer payments. Scholars have conducted extensive research on this topic. However, overall, existing studies generally adopt a unilateral perspective focused on local governments, primarily attributing the issue to moral hazard within local fiscal governance [38,39]. In reality, this outcome is the result of a negotiation between the two parties, with the central government providing some degree of support to local governments.

A review of relevant policies indicates that NCMS fiscal subsidies consist of both central transfer payments and local fiscal matching subsidies, with local governments responsible for fund expenditure management. In fact, transfer payments not only provide financial support from the central government for local public service delivery but also serve as a crucial fiscal instrument for central governance oversight of local authorities [40,41]. According to Qian and Cheng [42], when the central government provides more NCMS transfer payments to regions with lower fund balances, this can trigger strategic spending behavior by local governments. Specifically, local governments may curtail their collection and management efforts to maintain low fund balances and thereby secure additional transfer payments. However, under the dual incentives of economic decentralization and promotion mechanisms, central transfer payments provide local governments with considerable leeway in their strategies regarding shared responsibilities, leading to changes in local specialized expenditure behaviors. On the one hand, if local governments adopt cooperative strategies with the central government, direct funding via central special-purpose transfer payments tends to stimulate local fiscal matching subsidies for designated purposes. Consequently, local specialized expenditure increases in parallel with the growth of special-purpose revenues. On the other hand, local governments may also have motivations for non-cooperative strategies that benefit themselves. The imbalance between centralized revenue authority and decentralized expenditure responsibilities fosters a preference for productive expenditure among local governments. Self-interested behavior prompts local governments to excessively rely on central special-purpose transfer payments, treating them as readily available public funds. By shifting the costs of specialized expenditure onto the central government, local governments can divert matching subsidies intended for specialized uses to productive purposes without economic pressure [43,44]. Furthermore, specific mechanisms embedded in special-purpose transfer payments, including matching policies and infrastructure development, afford local governments the opportunity to covertly redirect originally designated matching subsidies for productive purposes.

In summary, existing studies have not reached a consensus on whether the NCMS can effectively reduce medical economic risks. Most studies asserting that the NCMS fails to provide adequate protection primarily attribute this to insufficient compensation spending. However, given the government’s continuous expansion of fiscal subsidies for the NCMS, it is not convincing to explain this solely by inadequate compensation spending. Moreover, there are few studies on the “central–local governments relationship” in the medical economic risks sharing, particularly regarding the strategic interactions between governments in NCMS fiscal subsidies. Building on the preceding discussion of strategic interactions between central and local governments regarding fiscal subsidies under shared responsibilities, this paper proposes the following hypothesis: If local governments adopt non-cooperative strategies with the central government concerning matching subsidies in response to NCMS transfer payments, the currently low reimbursement level may represent only a superficial explanation for the limited effectiveness of the NCMS in mitigating medical economic risks among insured rural households. The deeper underlying cause resides in the inefficient local fiscal matching subsidies induced by the complex intergovernmental strategic interactions that arise from NCMS transfer payments. To validate this hypothesis, the subsequent sections present both a theoretical and empirical analysis.

### 3.2. Theoretical Hypothesis

Within a fiscal decentralization system, the relationship between the central and local governments can be described as principal-agent dynamics. For shared responsibilities such as the NCMS, the central government’s primary objective is to induce reasonable matching subsidies from local governments via transfer payments, thereby ensuring that local NCMS compensation spending is aligned with NCMS fiscal subsidies. However, under the dual incentives of economic decentralization and promotion mechanisms, local governments may pursue strategic behaviors extending beyond merely providing matching subsidies in response to NCMS transfer payments. Specifically:

1. The central government implements a “full advance disbursement in the current year, with actual settlement in the following year” approach for NCMS transfer payments to local governments. Although any shortfall in local fiscal matching subsidies during the current year is deducted from future transfer payments at a certain ratio and requires local governments to make up the difference, this mechanism still provides local governments with some flexibility to divert some NCMS matching subsidies to productive uses. This is because the fiscal revenue generated from productive expenditure in the following year often exceeds the amount required to cover the matching subsidies.
2. According to annual settlement reports on NCMS transfer payments, certain regions experience reductions in transfer payments owing to untimely local fiscal matching subsidies. This indicates that the regulations exert only partial binding authority over local governments’ matching subsidies behaviors.
3. Given the increasing economic uncertainty, local governments encounter significant fiscal pressures, and central transfer payments can alleviate these financial difficulties to some extent. However, local governments may transfer the burden of NCMS matching subsidies onto central transfer payments.

Overall, because local NCMS compensation spending directly affects rural households’ medical spending decisions, if NCMS transfer payments generate both income and substitution effects on local fiscal matching subsidies, discrepancies between local fiscal matching subsidies and compensation spending may emerge. These discrepancies ultimately affect the expected medical expenditure of insured rural households, leading to increased uncertainty in their medical economic risks.

**Figure 1:**
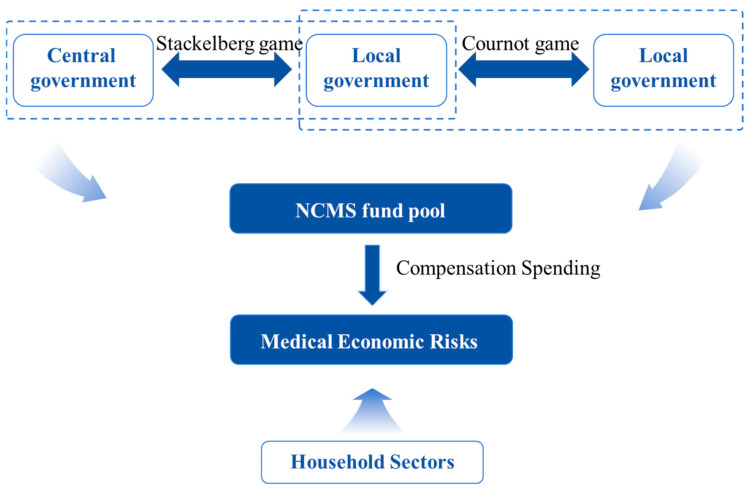
Dynamic game framework.

To clarify the impact of local fiscal matching subsidies on medical economic risks under the influence of transfer payments, we draw upon Ma’s [45] analysis of the strategic interactions between local governments and central transfer payments, combined with Grossman’s health demand model [46]. We develop a dynamic game framework encompassing the central government, local governments, and household sectors. As illustrated in Figure 1, first, the interaction between the central government and local governments is modeled as a Stackelberg game. The central government functions as the leader, initiating the transfer payments policy for the NCMS and allocating financial resources according to regional economic disparities. Local governments, as followers, must balance adherence to central government NCMS policies with the development of local economies when responding to transfer payments. They are expected to determine matching subsidy levels that optimize their own fiscal budgets. Second, interactions among local governments constitute a Cournot game, in which each local government operates as an independent decision-maker, simultaneously determining the scale of NCMS matching subsidies. Finally, the central government transfer payments and local fiscal matching subsidies collectively form the NCMS fund pool. This gaming process determines the scale of compensation spending, which directly affects reimbursement levels and out-of-pocket medical expenses.

Assuming that the government sector encompasses both central and local governments, local governments must strike a balance between adhering to the central NCMS policy and fostering the local economy. Local governments make matching subsidies decisions based on the scale of NCMS transfer payments provided by the central government to optimize their own fiscal budgets. Therefore, the interaction between local and central governments can be modeled as a Stackelberg game. The central government ideally expects local governments to provide matching funds in accordance with NCMS subsidy policies and adjusts transfer payments based on regional economic conditions and financial capabilities to reduce disparities in NCMS subsidies. Against this backdrop, the objective function of the central government is formulated as follows:

(1) $$\underset{\chi_{i}}{max}U_{c}=\ln(\chi_{1}S+y_{1})+\ln(\chi_{2}S+y_{2})$$

$$s.t.\;m_{1}\chi_{1}S+m_{2}\chi_{2}S\leq R$$

Among these, S denotes the annual NCMS subsidy standard, $\chi_{i}$ represents the central transfer payments coefficient, and $y_{i}$ refers to the local government matching subsidies. $m_{1}\chi_{1}S+m_{2}\chi_{2}S\leq R$ indicates the budget constraint condition for the central government’s NCMS transfer payments. $m_{i}$ and R respectively denote the NCMS enrollment population in region i and the fiscal budget allocated by the central government for NCMS transfer payments. It can be seen from Equation (1) that the central government makes separate decisions regarding NCMS transfer payments to two regions.

(2) $$\chi_{i}=\frac{m_{j}y_{j}+R-m_{i}y_{i}}{2m_{i}S}$$

Compared to the central government, which aims to maximize the utility of NCMS fiscal subsidies, local governments pursue the maximization of net revenue. In this context, the NCMS transfer payments $\chi_{i}S$ from the central government can only induce a partial matching subsidy $\gamma_{i}y_{i}$ from local governments. Local governments are more inclined to allocate the matching funds $\delta_{i}y_{i}$ to productive expenditure ($\gamma_{i}$ + $\delta_{i}$ = 1), thereby generating fiscal revenue $\theta_{i}y_{i}$. The local government’s NCMS fund expenditure in a given year is denoted by $E_{i}$, while the cost of fund supervision and productive input is represented by $\alpha_{i}[m_{i}(\chi_{i}S+\gamma_{i}y_{i}+\delta_{i}y_{i}+\beta_{i}y_{i}){]}^{2}$. Taking into account the NCMS transfer payments $\chi_{i}=\frac{m_{j}y_{j}+R-m_{i}y_{i}}{2m_{i}S}$ provided by the central government, the objective function of the local government can be expressed as:

(3) $$\underset{y_{i}}{max}U_{L}=m_{i}(\chi_{i}S+\gamma_{i}y_{i}+\beta_{i}y_{i}+\theta_{i}y_{i})-m_{i}E_{i}-\alpha_{i}[m_{i}(\chi_{i}S+\gamma_{i}y_{i}+\delta_{i}y_{i}+\beta_{i}y_{i}){]}^{2}$$

$$s.t.\;\chi_{i}=\frac{m_{j}y_{j}+R-m_{i}y_{i}}{2m_{i}S}$$

In the process of competing for NCMS transfer payments, the strategic interaction among local governments takes the form of a Cournot game, where each government determines its optimal matching subsidy level by taking the other’s optimal matching subsidy decision as given. From Equation (3), the optimal matching subsidy decisions of local governments can be derived as:

(4) $$y_{i}=\frac{\left(\gamma_{i}+\beta_{i}+\theta_{i}-1/2\right)}{2\alpha_{i}{\left(\gamma_{i}+\beta_{i}+\delta_{i}-1/2\right)}^{2}}-\frac{m_{j}y_{j}+R}{2m_{i}(\gamma_{i}+\beta_{i}+\delta_{i}-1/2)}$$

Following the household health investment model, the medical expenditure utility function for a representative rural household is specified as: $U_{i}=\sum_{t=1}^{n}[h_{it}^{1-\sigma }/(1-\sigma )+z_{it}^{1-\sigma }/(1-\sigma )]$, where $h_{it}$ denotes the rural households’ medical expenditure in period t, $z_{it}$ represents non-medical consumption expenditure, σ indicates the risk aversion coefficient.

Let $p_{it}$, $A_{it}$, $\beta_{it}$, and $B_{it}$ denote the price of medical services, rural household wealth, NCMS household premium rate, and local matching subsidy ratio, respectively. The budget constraint for rural household medical expenditure can then be expressed as: $p_{it}h_{it}+z_{it}\leq A_{it}+B_{it}y_{it}-\beta_{it}y_{it}$. Based on this utility function and budget constraint, combined with Equation (4), the optimal medical expenditure decision rule for rural households can be derived as:

(5) $$h_{i}^{*}=[A_{i}+(B_{i}-\beta_{i})y_{i}]/[p_{i}+p_{i}^{-\frac{1}{\sigma }}]$$

Given the current low NCMS reimbursement levels, an increase in reimbursement expenditure can enhance rural households’ expected medical expenditure, thereby reducing their medical economic risks. Building on this premise, the following hypotheses are derived from Equations (4) and (5):

Hypothesis 1: NCMS transfer payments induce partial local fiscal matching subsidies, which amplify NCMS reimbursement expenditure and consequently raise rural households’ expected medical expenditure ($[\partial h_{i}/\partial (B_{i}y_{i})][\partial (B_{i}y_{i})/\partial \gamma_{i}]>0$). This mechanism leads to a decline in medical economic risks.

Hypothesis 2: NCMS transfer payments displace part of the local fiscal matching subsidies, as local governments reallocate these funds toward productive expenditure. This reallocation crowds out NCMS reimbursement expenditure, thereby lowering rural households’ expected medical expenditure ($[\partial h_{i}/\partial (B_{i}y_{i})][\partial (B_{i}y_{i})/\partial \delta_{i}]<0$), which in turn increases medical economic risks. However, the returns generated by these productive investments may offset this effect by increasing NCMS reimbursement expenditure, thereby improving expected medical expenditure ($[\partial h_{i}/\partial (B_{i}y_{i})][\partial (B_{i}y_{i})/\partial \theta_{i}]>0$) and ultimately reducing medical economic risks.

## 4. Materials and Methods

### 4.1. Empirical Strategy

**Figure 2:**
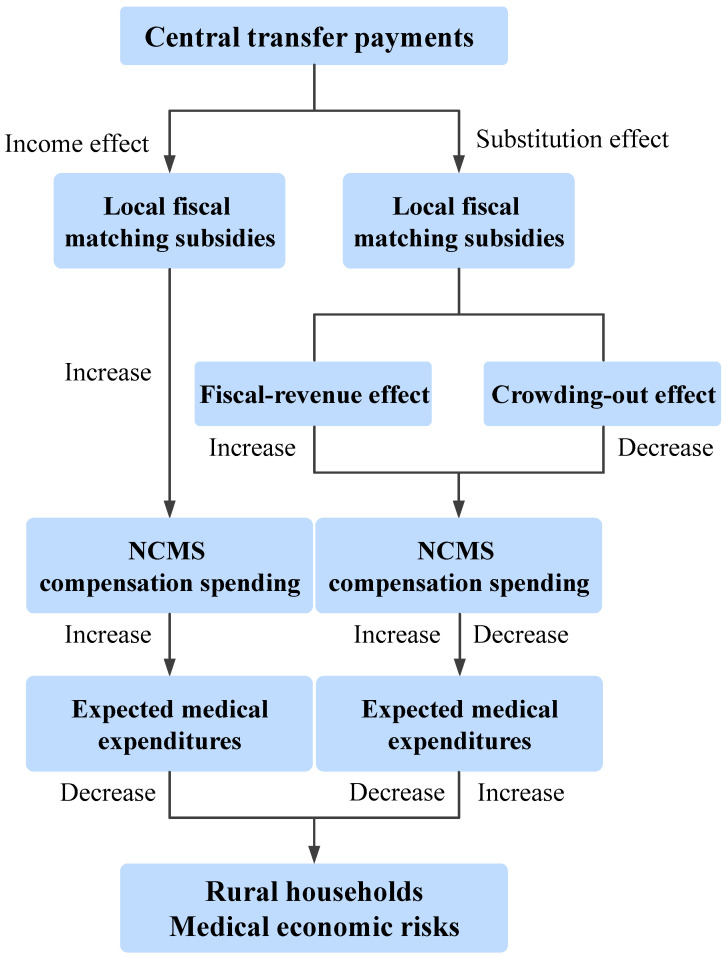
Influence mechanism framework.

Based on the derived hypotheses, local fiscal matching subsidies for the NCMS, moderated by central transfer payments, influence medical economic risks primarily through two pathways, as illustrated in Figure 2: (1) Central transfer payments prompt higher local fiscal matching subsidies for NCMS compensation, creating an income effect that raises rural households expected medical expenditure and thereby reduces their medical economic risks; (2) Local governments may shift part of the matching-subsidy burden onto these transfer payments and channel the freed-up funds into productive uses, crowding out NCMS compensation while the resulting productive returns generate a fiscal-revenue effect that can subsequently replenish NCMS compensation. Consequently, under the substitution effect of transfer payments, the overall impact of local fiscal matching subsidies on medical economic risks depends on the balance between the crowding-out effect and the fiscal-revenue effect.

To verify how local fiscal matching subsidies influence medical economic risks under the moderating effect of NCMS transfer payments, this study combines empirical regressions with a series of diagnostic checks. First, a panel Probit model estimates the impact of local fiscal matching subsidies on medical economic risks, considering the moderating effect of the NCMS transfer payments. Second, general transfer payments serve as an instrument for NCMS transfer payments to address potential endogeneity. Together, these steps provide an initial explanation of how the income and substitution effects of NCMS transfer payments influence the impact of local fiscal matching subsidies on medical economic risks. Further analyses include heterogeneity tests, robustness tests, and mechanism validation: group regressions by geographic location, secondary-industry development, and fiscal self-sufficiency assess whether regional disparities yield different results; robustness tests replace key variables and indicators and account for major policy shifts to confirm the stability of the baseline findings; and finally, in the mechanism test, NCMS compensation spending is introduced as a mediating variable to trace how local fiscal matching subsidies, under NCMS transfer payments, alter medical economic risks.

### 4.2. Variables and Data

#### 4.2.1. Dependent Variable

The dependent variable is an indicator of catastrophic health expenditure, which measures the risk of a household falling into poverty due to medical costs. Based on WHO’s 2003 publication HEALTH SYSTEMS PERFORMANCE ASSESSMENT: DEBATES, METHODS, AND EMPIRICISM [47], this paper defines medical expenditure as catastrophic when a household’s out-of-pocket payments are greater than or equal to 40% of its capacity to pay. Accordingly, if a household’s medical spending exceeds 40 percent of its non-food expenditure, the indicator equals 1; otherwise, it equals 0. Additionally, following Yazdi-Feyzabadi et al. [48], Kockaya et al. [49], Sun et al. [50], and Li et al. [51], alternative thresholds of 50% and 20% are used in the robustness tests. This measurement is consistent with rural China’s consumption characteristics: rural households have a high share of food expenditure in total consumption, and non-food expenditure more accurately reflects the actual disposable capacity for medical spending, thereby avoiding overestimation of catastrophic health expenditure. For robustness, this paper also redefines catastrophic health expenditure using total household expenditure and household income as alternative denominators.

#### 4.2.2. Core Explanatory, Moderating and Mediating Variables

The core explanatory variable is local fiscal matching subsidies, measured as local government NCMS contribution divided by the number of enrollees at year-end. The moderating variable is NCMS transfer payments, measured as the central government NCMS contribution divided by the number of enrollees at year-end. The mediating variable is NCMS compensation spending, measured as total NCMS reimbursements divided by the number of enrollees at year-end. This paper takes the natural logarithm of all three variables.

#### 4.2.3. Control Variables

Control variables include household member characteristics such as age, gender (1 for male, 0 for female), marital status (1 for unmarried, 0 for married), and hukou status (1 for agricultural hukou, 0 for non-agricultural hukou); household characteristics such as family size (number of household members), household health status (average health score of household members), household education level (average years of education of household members), social medical insurance coverage (proportion of household members covered by social medical insurance), and commercial health insurance coverage (proportion of household members covered by commercial health insurance); as well as regional characteristics such as fiscal revenue decentralization index (proportion of per capita local fiscal revenue to total per capita fiscal revenue), number of medical hospital beds per 1000 population, per capita GDP (natural logarithm), hospitalization rate, and aging level (proportion of the population aged 65 and above).

#### 4.2.4. Other Variables

Endogeneity is addressed by using general transfer payments as an instrument for NCMS transfer payments, measured as total central transfer payments divided by the population at year-end (natural logarithm). In heterogeneity tests, the fiscal-revenue decentralization index is replaced by indices of fiscal-expenditure decentralization (proportion of per capita local budget expenditure to total per capita budget expenditure) and fiscal autonomy (proportion of local budget revenue to local budget expenditure).

Descriptive statistics for all variables are presented in Table 1.

#### 4.2.5. Data Source

The household-level data used in this study are sourced from CFPS for the period from 2010 to 2018. The data on NCMS transfer payments, local fiscal matching subsidies and NCMS compensation spending are obtained from the Statistical Handbook of the New Rural Cooperative Medical Scheme Information for the years 2008–2015. The alignment of micro-level and macro-level data across different years is justified by three key considerations: First, theoretical hypotheses derived from the model suggest that local fiscal matching subsidies, under the moderating effect of NCMS transfer payments, influence rural households’ expected medical expenditure rather than current expenditure via compensatory spending. Second, for rural households, reimbursement events occur only when hospitalization expenses arise; consequently, the probability that NCMS fiscal subsidies to rural households are converted into compensatory expenditure in the same funding year remains low. Third, the NCMS transfer payments mechanism operates on a “pre-allocation in the current year and settlement in the following year” system for local disbursements, resulting in a temporal lag in the moderating effect of NCMS transfer payments on local fiscal matching subsidies. Collectively, these factors indicate that local fiscal matching subsidies exhibit a delayed impact on medical economic risks, a phenomenon further amplified by the moderating effect of NCMS transfer payments. Additionally, the CFPS collects data biennially, whereas the Statistical Handbook of the New Rural Cooperative Medical Scheme Information provides annual statistics, enabling the integration of micro- and macro-level datasets. The Statistical Handbook ceased publication in 2015 due to the gradual transition of regional NCMS programs to urban-rural integrated schemes beginning in 2016. Notably, some provincial data for specific years (e.g., 2009 and 2010) contain missing values. Based on this, to minimize the temporal misalignment between the two datasets and account for the aforementioned circumstances, this paper aligns macro-level data from 2008, 2011, 2013, 2014, and 2015 with micro-level data from 2010, 2012, 2014, 2016, and 2018, respectively. The empirical sample consists of 87,630 individuals in total, excluding those with missing variable values.

### 4.3. Model Selection

Due to the binary nature of the dependent variable, employing traditional OLS regression methods would result in biased parameter estimates. Given this, referring to previous studies [52,53], this paper employs a panel Probit model based on the cumulative normal distribution function to account for the binary characteristics. Additionally, to examine the moderating effect of NCMS transfer payments on local fiscal matching subsidies, an interaction term is included, following Liu and Zhang [54] and Cui et al. [55]. The interaction term identifies the heterogeneous impact of local fiscal matching subsidies on medical economic risks under different levels of central transfer payments, which aligns with the theoretical hypotheses of the income and substitution effect. The econometric model is specified as follows:

(6) $$C=\alpha_{0}+\alpha_{1}S+\alpha_{2}X+\varepsilon$$

(7) $$C=\alpha_{0}+\alpha_{1}S+\alpha_{2}T+\alpha_{3}S\times T+\alpha_{4}X+\varepsilon$$

In this context, C denotes whether a rural household incurs catastrophic health expenditure, S represents the local fiscal matching subsidies, T refers to the central government’s NCMS transfer payments, and S×T captures the interaction between the local fiscal matching subsidies and the central NCMS transfer payments to the same region, while X denotes a vector of control variables and ε accounts for the stochastic error term.

For the robustness tests addressing major reforms in the medical insurance system and the healthcare sector, a panel Probit difference-in-differences (DID) model is employed to capture the policy impact. Specifically, by comparing the changes between the treatment and control group before and after the policy implementation, the DID model isolates the net effect of reform from other confounding factors. This method effectively addresses potential endogeneity arising from concurrent policy reforms. The econometric framework is specified as follows:

(8) $$C=\alpha_{0}+\alpha_{1}S+\alpha_{2}T+\alpha_{3}S\times T+\alpha_{4}policy+\alpha_{5}time+\alpha_{6}did+\alpha_{7}X+\varepsilon$$

In this context, policy indicates whether a policy change occurred (1 = yes, 0 = no), and time represents the policy-change year (1 for the year of the change and subsequent years, 0 otherwise). did is the interaction term between policy and time (equal to 1 when both policy and time are 1, and 0 otherwise), used to estimate the impact of major reforms in the medical insurance system and healthcare system on medical economic risks.

In the mechanism analysis, the aim is to examine how local fiscal matching subsidies, under the moderating effect of NCMS transfer payments, influence rural households’ expected medical expenditure through NCMS compensation spending, thereby affecting their medical economic risks. On the one hand, central transfer payments and local fiscal matching subsidies constitute the primary sources of the NCMS fund pool; an increase in these funds directly expands the fund’s scale and raises the per capita compensation spending level. Conversely, a reduction subjects the NCMS fund pool to contractionary pressure, as declining total fund revenues force a reduction in compensation spending. On the other hand, higher compensation spending enables insured individuals to receive greater reimbursement, which directly reduces out-of-pocket expenses and alleviates the financial burden of healthcare. Conversely, lower compensation spending implies that insured patients must bear a higher proportion of medical expenses, increasing the likelihood of catastrophic health expenditures. Thus, central transfer payments and local fiscal matching subsidies influence medical economic risks through local compensation spending, indicating that NCMS compensation spending acts as a mediating variable in this mechanism. To test this mediation effect, the study follows the approach proposed by Baron and Kenny [56] and draw on other studies [57], conducting a three-step regression procedure: first, regress the dependent variable on the core explanatory variables; second, regress the mediating variable on the core explanatory variables; and finally, regress the dependent variable on both the core explanatory variables and the mediating variable. The specific econometric models are specified as follows:

(9) $$C=\alpha_{0}+\alpha_{1}S+\alpha_{2}T+\alpha_{3}S\times T+\alpha_{4}X+\varepsilon$$

(10) $$CS=\alpha_{0}+\alpha_{1}S+\alpha_{2}T+\alpha_{3}S\times T+\alpha_{4}X+\varepsilon$$

(11) $$C=\alpha_{0}+\alpha_{1}S+\alpha_{2}T+\alpha_{3}S\times T+\alpha_{4}CS+\alpha_{5}X+\varepsilon$$

where CS represents NCMS compensation spending, which serves as the mediating variable.

All regressions incorporate the corresponding CFPS sampling weights and include two-way fixed effects for region and time.

## 5. Results

### 5.1. Basis Regression Results and Endogeneity Discussions

**Figure 3:**
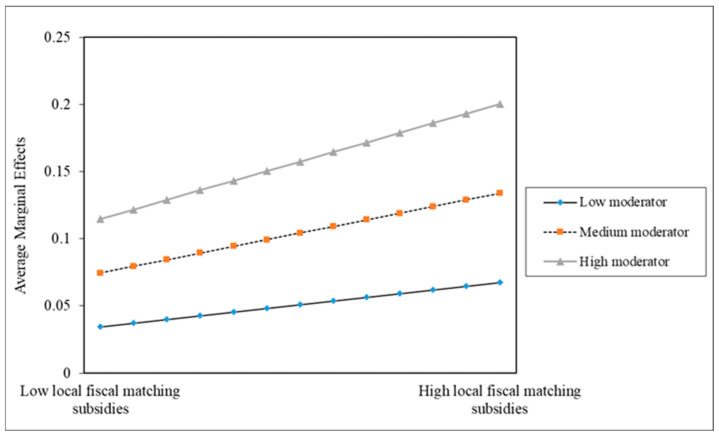
Simple slope plot at different levels of NCMS transfer payments.

The basis regression results show that, in Column 1 of Table 2, when only the core explanatory variable is examined, the logarithm of per capita local fiscal matching subsidy is significantly negative at the 1% statistical level, with an average marginal effect of −0.0204. This indicates that a 1% increase in local fiscal matching subsidies is associated with an average decline of 0.0204 percentage points in the probability of catastrophic health expenditure. This finding suggests that local fiscal matching subsidies significantly reduce catastrophic health expenditure among rural households under the income effect of NCMS transfer payments. In Column 2 of Table 2, local fiscal matching subsidies are significantly negatively associated with catastrophic health expenditure in rural households, while the interaction term between local fiscal matching subsidies and NCMS transfer payments is significantly correlated with catastrophic health expenditure. Given the non-linear nature of the Probit model, the regression coefficient of the interaction term cannot be directly interpreted as the true marginal effect of the moderating effect. This paper further decomposes the marginal effect of the interaction term: the average marginal effects of local fiscal matching subsidies on catastrophic health expenditure are calculated at three critical points of NCMS transfer payments, namely the mean, the mean minus one standard deviation and the mean plus one standard deviation. The results show that when NCMS transfer payments are at a low level (mean minus one standard deviation), the marginal effect of local fiscal matching subsidies is −0.0157, indicating that local fiscal matching subsidies have a stronger effect in reducing catastrophic health expenditure. When NCMS transfer payments are at a high level (mean plus one standard deviation), the marginal effect of local fiscal matching subsidies is −0.0128, and this relationship becomes weaker (as shown in Figure 3).

These findings indicate that, under the joint moderating effects of income and substitution effects, local fiscal matching subsidies have differential impacts on catastrophic health expenditure. Specifically, under the income effect, local fiscal matching subsidies significantly reduce catastrophic health expenditure in rural households, whereas under the substitution effect, they significantly increase it. In other words, local governments adopt a non-cooperative strategic response to NCMS transfer payments, thereby introducing uncertainty into medical economic risks. From the perspective of policy implementation, this finding provides guidance for optimizing the NCMS fiscal subsidies policy. On the one hand, the design of local fiscal matching subsidies should be dynamically adjusted according to the relative strengths of the income and substitution effects. For example, specifying the designated uses of matching subsidies can strengthen the positive impact of the income effect and curb the negative impact of the substitution effect. On the other hand, the moderating role of central transfer payments should be appropriately leveraged. The scale of transfer payments where local fiscal matching subsidies demonstrate high actual utilization efficiency should be expanded, guiding local governments to prioritize safeguarding livelihood expenditures over short-term productive gains. These policy adjustments would effectively reduce uncertainties arising from local strategic behavior and enhance the practical effectiveness of the NCMS in mitigating catastrophic health expenditure for rural households.

Considering that changes in the incidence of catastrophic health expenditure among rural households may inversely influence adjustments in local fiscal matching subsidies, this study potentially faces bidirectional causality between the dependent and independent variables. Although the use of lagged variables for both NCMS transfer payments and local fiscal matching subsidies has mitigated endogeneity-induced bias to some extent, we further address this issue by employing a two-stage Instrumental Variable Probit model, using general transfer payments as the IV for local fiscal matching subsidies and adhering to the exclusion restriction principle. A valid instrumental variable must satisfy two key conditions: relevance and exogeneity. As outlined in the institutional background, NCMS transfer payments are classified as general transfer payments but exhibit characteristics of special-purpose transfer payments. This institutional arrangement creates a budgetary linkage between general transfer payments and local fiscal matching subsidies. Specifically, when the central government increases general transfer payments, local governments’ fiscal capacity expands, thereby enhancing their ability to provide matching subsidies. This indicates that general transfer payments exert a significant influence on local fiscal matching subsidies, demonstrating their strong relevance. Additionally, unlike special-purpose transfer payments, the primary function of general transfer payments is to balance fiscal disparities across regions and compensate for the fiscal gap arising from insufficient resource endowments. The allocation of these funds is determined autonomously by local governments and is not directly tied to specific healthcare items or services. For the NCMS, medical insurance fiscal subsidies are included in “joint fiscal powers transfer payments”, with expenditure responsibility resting with local governments. Through the processes of local budgetary decision-making, local fiscal matching subsidies, and the medical insurance fund pool, general transfer payments ultimately influence medical economic risks. It follows that general transfer payments are excluded from directly affecting medical economic risks. In summary, using general transfer payments as an instrumental variable for local fiscal matching subsidies is theoretically justified.

The results of the two-stage IV-Probit model indicate that the Wald test for exogeneity yields a *p*-value of 0.000, leading to the rejection of the null hypothesis and confirming the presence of endogeneity. The weak instrument test shows that both the AR and Wald statistics yield *p*-values of 0.000, thereby rejecting the null hypothesis that the endogenous variable is uncorrelated with the instrument variable. Collectively, these findings validate the use of general transfer payments as a valid and reliable instrumental variable. In addition, the results in Columns (3) and (4) of Table 2 remain consistent with the basis regression, confirming the robustness of our findings after addressing endogeneity.

### 5.2. Robustness Tests

Robustness Test 1: Alternative Measures of Catastrophic Health Expenditure. The WHO typically defines households with catastrophic health expenditure as those spending more than 40% of non-food expenditure on healthcare. However, due to cross-country heterogeneity in economic conditions and regional disparities in resource endowments, scholars have not reached a consensus on the measurement standards for catastrophic health expenditure. With reference to previous studies, we adopt two alternative CHE thresholds (20% and 50%) and denominators (household expenditure and household income) for robustness tests [58,59]. Table 3 shows that the basis results remain consistent across these alternative specifications.

Robustness Test 2: Alternative Fiscal Decentralization Measures. The moderating effect of NCMS transfer payments on local subsidies operates within China’s fiscal decentralization framework. Fiscal decentralization metrics can be categorized into fiscal revenue decentralization and fiscal expenditure decentralization (reflecting local reliance on central funds), and fiscal autonomy (capturing local fiscal capacity). Divergent measures may introduce estimation bias. Drawing on existing literature, we test the robustness using expenditure decentralization and fiscal autonomy indices [60,61]. As shown in Table 4, the results maintain consistent significance levels and coefficient signs compared to the basis regression.

Robustness Test 3: Excluding Exogenous Policy Shocks. This paper considers two policy shocks: the integration of the urban and rural medical insurance systems in 2016, and the healthcare system reform in 2018. Specifically, in 2016, the central government mandated local authorities to implement the urban-rural integration policy for basic medical insurance. Currently, fiscal subsidies constitute a substantial proportion of funding in the urban residents’ basic medical insurance system. Consequently, the integration of urban and rural basic medical insurance undoubtedly increases fiscal pressure on local governments, potentially affecting the moderating role of NCMS transfer payments on local fiscal matching subsidies and subsequently influencing medical economic risks of rural households. Using 2016 as the cutoff year, we divided the regions into treatment groups (those that had implemented the integration) and control groups (those that had not implemented the integration). Furthermore, in July 2018, the State Council issued the “Notice on the Reform Plan for Division of Central and Local Financial Responsibilities and Expenditure Obligations in the Healthcare Sector”. The implementation of this policy affects the bargaining relationship between central and local governments regarding NCMS fiscal subsidies, making the moderating effect of NCMS transfer payments on local fiscal matching subsidies more complex. This complexity may lead to fluctuations in rural households’ medical expenditure decisions and consequently increase uncertainty in their medical economic risks. Using 2018 as the cutoff year, we categorized samples into treatment and control groups based on reform implementation status. To exclude exogenous policy shocks, this paper employs a panel Probit DID model for further regression analysis. As evident from Table 5, the empirical results remain highly robust compared to the basis regression, demonstrating that the conclusions are not biased by the integration of urban and rural medical insurance systems and the healthcare system reform.

Robustness Test 4: Alternative Lag Structures. This paper matches annual provincial fiscal variables to subsequent CFPS waves to capture lagged policy effects. However, because CHE is constructed based on household expenditure during the 12 months preceding the survey, and fiscal variables are measured as annual aggregates, a partial overlap between fiscal exposure and the recall window of expenditure may arise in some matching years. Although fiscal variables are interpreted as institutional funding environment indicators rather than point-in-time shocks, such overlap could potentially introduce measurement attenuation. To address this issue, this paper implements a stricter lag specification in which fiscal variables precede CHE measurement by more than one survey wave. Specifically, robustness test 4 matches 2008 fiscal data to the 2010 CFPS wave, 2011 fiscal data to the 2014 CFPS wave, 2013 fiscal data to the 2016 CFPS wave, and 2014 fiscal data to the 2018 CFPS wave. Similar to Table 2, Columns (1) and (2) in Table 6 present the basis regression results, while Columns (3) and (4) display the endogeneity test results. It can be found that the signs and significance levels of the coefficients for the core variables are largely consistent with those in Table 2, confirming that the main conclusions are not driven by potential overlap in measurement windows.

Robustness Test 5: Assessing Omitted Variable Bias. Although the basis specifications incorporate multiple dimensions of control variables and two-way fixed effects, it is difficult to fully rule out omitted variable bias. To quantitatively assess the robustness to potential unobserved confounding, this paper implements two complementary sensitivity analyses. First, following Oster [62], this paper applies a bounding approach to evaluate the potential magnitude of selection on unobservables relative to observables. This method compares coefficient movements and changes in R^2^ across nested model specifications to compute the parameter δ, which measures the degree of selection on unobservables required to fully attenuate the estimated effect. A δ value greater than 1 indicates that selection on unobservables would need to be at least as strong as selection on observables to eliminate the estimated coefficient, suggesting substantial robustness. As the method is derived under linear regression assumptions, this paper re-estimates the core model using a linear probability model (LPM) and compute δ accordingly. Second, this paper computes the E-value to quantify the minimum strength of association that an unmeasured confounder would need to have with both the explanatory variables and the dependent variable in order to fully explain away the observed effect. Larger E-values imply that only an unobserved confounder with implausibly strong associations could overturn the findings. The E-value is primarily calculated based on the risk ratio (RR) or odds ratio (OR). In the basis regression, the Probit model is applied. By examining marginal effects, the RR is obtained, yielding E-values of 1.7150 and 1.5361, respectively. This indicates that the conclusions remain valid even after controlling for omitted variable bias. In the robustness test, this paper re-estimates the core specification using a Logit model and calculates the corresponding E-values based on the OR. According to Table 7, both δ and the E-value are greater than 1, which further verifies the robustness of the main findings.

Robustness Test 6: Relaxed Exclusion Restrictions. Although the basis analysis discusses the relevance and exclusion restrictions of the instrumental variable, prior research suggests that general transfer payments may influence household catastrophic health expenditure through broader fiscal capacity and expenditure allocation channels, raising concerns about the strict exogeneity assumption of the instrument [63]. To address this issue, this paper implements the plausibly exogenous framework proposed by Conley et al. [64], which relaxes the exclusion restriction by allowing the instrument to exert a small direct effect on the dependent variable. Specifically, this paper applies the Union of Confidence Intervals (UCI) approach to examine the robustness of the estimation results when the instrument is not fully exogenous. The results in Table 8 show that the confidence interval for the coefficient of the core explanatory variable does not contain zero. Therefore, under the plausibly exogenous condition, the IV estimates remain robust.

Robustness Test 7: Selection Bias and Missing Data. In the 2010–2018 CFPS, the target population includes 128,934 observations. After excluding samples with missing values for all control variables, the final analysis sample consists of 87,630 observations. Non-random missing values could potentially introduce sample selection bias, leading to biased and inconsistent estimates. To address this concern, this paper conducts a balance check by comparing the 87,630 samples included in the analysis with those excluded due to missing data.

The results in Table 9 indicate that the two groups only exhibit significant differences in hukou status and family size, with no significant differences in the dependent variable or key explanatory variables. To further mitigate the potential impact of non-random missing values on the conclusions, this paper performs a robustness test using inverse probability weighting (IPW). First, a Probit selection equation is constructed with observable characteristics as independent variables and “sample inclusion status” as the dependent variable to compute IPW. Then, the weighted sample is used to re-estimate the basis model, correcting for potential sample selection bias.

As shown in Table 10, the coefficients and statistical significance of the key explanatory variables are consistent with the basis regression results. This confirms that the core conclusions are not affected by potential sample selection bias due to non-random missing values.

### 5.3. Heterogeneity Tests

This paper incorporates multi-group comparisons to examine regional differences in the impact of local fiscal matching subsidies on catastrophic health expenditure. First, the NCMS operates under a regionally differentiated fiscal sharing rule between the central and local governments. The central government provides a substantially higher transfer payment ratio to central and western rural regions than to eastern provinces, while eastern provinces are required to bear a larger share of local matching responsibilities. This institutional arrangement directly shapes local governments’ incentives regarding fiscal matching subsidies. Accordingly, the sample is divided into eastern and central/western regions for separate estimations. Second, fiscal self-sufficiency serves as a key indicator of local fiscal autonomy, reflecting the degree of reliance on central transfers and the flexibility in allocating expenditures between welfare and productive spending. Provinces with higher fiscal self-sufficiency face relatively softer budget constraints and enjoy greater discretion in determining fiscal matching subsidies for the NCMS, whereas provinces with lower fiscal self-sufficiency depend more heavily on central transfers and possess more limited fiscal adjustment space. Based on this reasoning, the sample is further divided into high and low fiscal self-sufficiency groups. Third, under the “promotion tournament” incentive system in China, local governments often exhibit a structural bias toward productive expenditures over welfare spending. The secondary industry plays a central role in driving local GDP growth and fiscal revenue. Regions with more developed secondary industries may therefore face stronger incentives to prioritize productive investments, potentially creating competition for fiscal resources allocated to welfare programs such as the NCMS. The sample is thus divided into regions with more and less developed secondary industries for additional subgroup analysis.

Table 11 shows that across all regional subgroups, local fiscal matching subsidies significantly reduce catastrophic health expenditure under the income effect of NCMS transfer payments, while significantly increasing such expenditure under the substitution effect. Notably, the coefficients for local fiscal matching subsidies, NCMS transfer payments and the interaction term are larger in eastern regions than in central and western regions, in areas with more developed secondary industry compared to less developed ones, and in regions with higher fiscal self-sufficiency rates relative to those with lower rates. This pattern arises because the economically developed and fiscally affluent eastern regions inherently maintain higher fiscal expenditure across all sectors than their less developed and fiscally constrained central and western counterparts. As a result, local fiscal matching subsidies exert a more pronounced impact of local fiscal matching subsidies on catastrophic health expenditure under the moderating effects of NCMS transfer payments.

### 5.4. Mechanism Tests

As proposed earlier, local fiscal matching subsidies primarily influence catastrophic health expenditure through two pathways under the moderating effect of central transfer payments. Building on the basis regression, this study employs a mediation effect model to examine the underlying mechanisms. The results in Table 12 indicate the following. First, NCMS transfer payments induce local fiscal matching subsidies to generate an income effect on NCMS compensation spending, which increases expected medical expenditure among insured rural households and consequently reduces their incidence of catastrophic health expenditure. Second, local governments reallocate part of the NCMS matching subsidy costs to transfer payments for productive expenditures. In this case, the substitution effect on NCMS compensation spending outweighs the income effect generated by productive benefits, thereby reducing expected medical expenditure while increasing catastrophic health expenditure. In conclusion, under these combined mechanisms, NCMS compensation spending reduces insured rural households’ expected medical expenditure while increasing their catastrophic health expenditure. This finding confirms our initial hypothesis: inadequate compensation merely reflects the NCMS’s superficial inability to effectively mitigate medical economic risks. The deeper explanation lies in the inefficient local fiscal matching subsidies, which result from the complex game-theoretic strategies between local and central governments in response to NCMS transfer payments.

## 6. Discussion

This study employs theoretical modeling and econometric analysis to demonstrate that inadequate compensation, acting as an external factor, undermines the NCMS’s effectiveness in mitigating medical economic risks. The root cause is the inefficient allocation of matching subsidies, which arises from complex local game-theoretic responses to central government transfer payments. Existing studies on China’s NCMS reveal significant divergences: some emphasize that the NCMS substantially reduces catastrophic health expenditure through risk sharing, whereas others argue that inadequate compensation constrains its protective effects. Nevertheless, these studies similarly tend to treat NCMS fiscal subsidies as an exogenous policy input, while ignoring the endogenous strategic interaction between central and local governments in the allocation of medical insurance fiscal funds. The absence of this theoretical perspective is likely to have led to contradictory conclusions in existing research. This paper offers a novel interpretive framework for this academic controversy by differentiating between the “income effect” and the “substitution effect” of local fiscal matching subsidies. Theoretical analysis suggests that when local fiscal matching subsidies increase due to incentives from central transfer payments (i.e., dominated by the income effect), the expansion of the NCMS fund pool directly boosts compensation spending. This, in turn, enhances the management of rural households’ expected medical expenditure, aligning with the prevailing conclusion that “the NCMS effectively disperses health risks”. However, when local governments allocate more fiscal funds to productive expenditures (dominated by the substitution effect), compensation spending is crowded out, ultimately raising households’ out-of-pocket burdens, which corresponds to the criticism of “insufficient compensation”. Empirical results indicate that the net outcome of these two effects depends on their relative intensity. Under the current fiscal structure, the negative impact of the substitution effect predominates, explaining why some studies report limited protection from the NCMS. This study reveals that the key constraint on the effectiveness of the NCMS lies not in the compensation system itself but in the strategic behavior of local governments, thereby broadening the analytical perspective of related research.

The game-theoretic strategies between central and local governments stem from the combined effects of China’s fiscal decentralization and political promotion incentive system. Following the tax-sharing reform, local officials faced strong incentives in an “economic growth competition,” where quantifiable metrics such as GDP growth rates and fiscal revenue became the core criteria for promotion. This led to a systemic bias in the allocation of public resources by local governments, favoring productive expenditures over welfare expenditures. As a type of welfare expenditure, the NCMS matching subsidies yield far lower short-term economic returns than productive investments. Consequently, local governments are reluctant to allocate their limited fiscal resources to long-term healthcare protection. When the cost of matching subsidies exceeds the benefits, local governments may strategically reduce their contributions, thereby undermining rural households’ access to healthcare protection. It can be seen that if the central government merely imposes mandatory matching requirements without establishing binding incentive mechanisms governing the actual use of matching funds, such arrangements may induce substitution behavior at the local level. This helps explain why increases in fiscal input have not generated uniform improvements in healthcare protection. Therefore, policy design should combine fiscal support with performance-based evaluation and transparency mechanisms to ensure that central funds are effectively translated into household benefits. However, it is important to emphasize that inefficient local fiscal matching subsidies should not be automatically interpreted as evidence of passive attitudes or intentional underperformance by local governments. In regions with weaker fiscal capacity or relatively high public health expenditure burdens, resource reallocation may stem from binding fiscal constraints rather than purely strategic substitution. Accordingly, this study emphasizes the role of incentive structures in shaping behavioral boundaries, rather than attributing outcomes solely to local government motives.

The game-theoretic strategies in China are not an isolated case. Mismatches between central transfer payments and local implementation are common in decentralized systems worldwide, but differences in institutional designs across countries produce divergent outcomes. For example, in India, the central government transfers payments to state governments through the National Health Mission (NHM), yet some states prioritize allocating these resources to infrastructure development to stimulate short-term economic growth [65]. Similarly, Brazil’s Unified Health System (SUS) relies on state and municipal governments for matching funds, yet local governments frequently reduce investments in primary healthcare because of fiscal pressures [66]. These cases share a common feature: under decentralized systems, local governments face “multiple conflicting goals”. In other words, they must implement central government welfare policies while simultaneously pursuing local economic growth. It is also worth noting that countries differ substantially in the degree of fiscal decentralization and budget constraints. Consequently, the “income effect and substitution effect” analytical framework developed in this study is not universally applicable without qualification. It possesses stronger explanatory power in decentralized systems similar to China, where top-down political incentive structures significantly influence local government behavior. In countries operating under different institutional arrangements, the strategic interaction between central and local governments in allocating medical insurance funds may follow distinct dynamics.

## 7. Conclusions

A review of the relevant literature reveals that most existing studies attribute the limited reduction in medical economic risks under the NCMS to inadequate compensation spending. However, given the continuous increase in government fiscal subsidies, attributing the problem solely to inadequate compensation spending is unconvincing. Moreover, few studies have analyzed the direct impact of NCMS on medical economic risks from the perspective of the central–local fiscal subsidies relationship. In fact, since the establishment of the NCMS, the central government has required local governments to provide matching subsidies in response to NCMS transfer payments. As central transfer payments to local governments for the NCMS have continued to rise, the moderating effect of these transfer payments on local fiscal matching subsidies has also intensified. Under the current fiscal decentralization system, central earmarked transfer payments are prone to inducing strategic behavior by local governments in their matching subsidies. This context raises the central research question: Do NCMS transfer payments trigger strategic behavior in local fiscal matching subsidies, thereby creating a misalignment between local subsidy practices and NCMS compensation outcomes, and ultimately influencing the medical economic risks of insured rural households? Accordingly, this paper addresses these issues from both theoretical and empirical perspectives.

Overall, this paper investigates why the NCMS struggles to deliver its protective benefits effectively, offering a novel perspective that enriches the existing literature. Furthermore, the findings yield valuable insights for fiscal and healthcare policy reforms in countries with contexts similar to China’s. Theoretical analysis demonstrates that: (1) NCMS transfer payments induce local fiscal matching subsidies to generate an income effect for NCMS compensation spending, thereby raising rural households’ expected medical expenditure and reducing their medical economic risks; (2) local governments divert part of the matching subsidy costs into productive uses by relying on NCMS transfer payments, thereby crowding out NCMS compensation spending. While such productive expenditures increase NCMS-related fiscal revenue, under the substitution effect, the impact of local fiscal matching subsidies on medical economic risks depends on the relative magnitudes of the substitution and income effects. Empirical analysis further confirms that local fiscal matching subsidies significantly reduce medical economic risks under the income effect but significantly increase them under the substitution effect. This alignment with our theoretical hypotheses simultaneously clarifies that inadequate compensation merely represents the superficial reason for NCMS’s limited risk mitigation capacity, with the root cause being inefficient matching subsidies resulting from local governments’ complex game-theoretic strategies in response to central NCMS transfer payments.

## 8. Policy Suggestions

Based on the above theoretical and empirical findings, this paper proposes the following policy recommendations. First, it is essential to clarify the boundaries of local fiscal matching subsidies. To address the strategic behavior of local governments diverting matching funds toward productive purposes, the central government should formulate explicit rules for matching subsidies that reflect differentiated fiscal capacities. For example, counties could be categorized into “high fiscal capacity,” “medium fiscal capacity”, and “low fiscal capacity” groups according to indicators such as per capita GDP and public budget revenue. Correspondingly, binding regulations should mandate a minimum matching ratio for high-capacity groups and impose a ceiling on the matching ratio for low-capacity groups. Furthermore, local governments must be strictly prohibited from reallocating NCMS matching subsidies to non-healthcare uses such as infrastructure projects or industrial support. Audit mechanisms should be implemented to trace fund flows, and any evidence of misallocation into non-healthcare accounts should lead to a reduction in the subsequent year’s central transfer payments. Second, a performance-oriented moderating mechanism for central transfer payments should be established. The central government should base the allocation of central transfer payments on the actual efficiency of local fiscal matching subsidies. For instance, indicators such as the share of NCMS compensation spending in total fund expenditures and the reduction rate in the incidence of catastrophic health expenditure could be used to calculate a composite performance score for each locality. Regions with higher performance should receive increased central transfer payments in the following year. In comparison, regions with lower performance should face reductions in their subsequent allocations. Third, transfer payments for urban and rural residents’ medical insurance should be regulated in line with the principles of general transfer payments. In coordinating basic medical insurance for urban and rural residents, the central government should apply the principles of general transfer payments to regulate the management of medical insurance transfer payments. This approach should effectively narrow the financing gap in medical insurance between urban and rural residents across regions and reduce the occurrence of regional medical economic risks. For example, minimum financing ratios for urban and rural residents’ medical insurance could be specified at the central, provincial, and county levels, with any local funding gap covered through general transfer payments to minimize disparities between urban and rural financing levels. In addition, financing ratios should be automatically adjusted on an annual basis in accordance with indicators such as regional per capita disposable income growth and medical expenses growth, thereby preventing rural households from bearing an excessive financial burden caused by economic fluctuations.

The study has several limitations. On the one hand, the theoretical analysis primarily examines the game strategies between local and central governments regarding NCMS fiscal subsidies but pays insufficient attention to household behavior. However, in practice, household expectations of government behavior under fiscal information asymmetry may also deviate, which requires more complex and systematic investigation. On the other hand, the empirical analysis relies on provincial-level data, which constrains analytical precision. First, the actual responsibility for managing medical insurance rests primarily with city and county governments, which face more immediate fiscal pressures and stronger public demands. Consequently, their financial conditions and policymaking exert a substantial influence on the effectiveness of medical insurance implementation. However, provincial data only reflects an “average level”, thereby obscuring disparities among sub-provincial governments. Second, although provincial transfer payments to cities and counties generally follow patterns similar to those of central-to-provincial transfer payments, cities and counties face greater fiscal constraints than provincial governments and display stronger non-cooperative behaviors in providing matching subsidies. The above is difficult to identify and analyze accurately using provincial-level data. Third, existing research indicates that there exists a spatial variability between administrative reimbursement boundaries and the actual coverage of medical services in rural China [67]. When provincial disparities in healthcare accessibility are not controlled for, the use of provincial-level fiscal data may introduce measurement errors, thereby affecting the precision of estimation results. Additionally, due to the dual limitations of the publication of NCMS data and the survey period of the CFPS, this study is only able to match the 2008 provincial fiscal data with the 2010 CFPS wave in the basis analysis. This matching approach fails to capture the dynamic changes in NCMS fiscal policies during the household medical expenditure recall window, inevitably introducing classical measurement error, which may lead to attenuation bias in the coefficient estimates, pushing the results towards null.

Based on the above limitations, future research can further explore the impact of medical insurance fiscal policies on different household groups, with a particular focus on vulnerable groups. In addition, municipal and county-level data can be utilized and precisely matched with household medical expenditure timepoints to better capture policy dynamics during the relevant period.

## Figures and Tables

**Table 1:** Descriptive statistics of variables.

Variable	Sample Size	Mean	Standard Deviation	Minimum	Maximum
Catastrophic health expenditure	87,630	0.1174	0.3218	0	1
Local fiscal matching subsidies	87,630	4.1685	0.5034	2.4650	5.2510
NCMS transfer payments	87,630	4.1187	1.3371	0	6.0509
Interaction term	87,630	8.1987	1.7853	0	10.9085
NCMS compensation spending	87,630	5.1249	0.4927	3.7003	7.0436
Age	87,630	52.3696	14.7880	16	90
Gender	87,630	0.5010	0.5000	0	1
Marital status	87,630	0.7197	0.4492	0	1
Hukou status	87,630	0.9320	0.2518	0	1
Family size	87,630	4.6048	1.9313	1	19
Household health status	87,630	2.7058	1.1118	1	5
Household education level	87,630	2.0067	1.7969	0	22.5000
Social medical insurance	87,630	0.7092	0.2232	0.0714	1
Commercial health insurance	87,630	0.0059	0.0567	0	1
Fiscal revenue decentralization	87,630	0.4402	0.0993	0.3010	0.8350
Beds	87,630	4.8619	1.0541	2.5100	7.4400
GDP	87,630	10.4745	0.4347	9.2408	11.8509
Hospitalization	87,630	0.1445	0.0335	0.0812	0.2260
Aging	87,630	0.1008	0.0184	0.0498	0.1499
General transfer payments	87,630	5.1249	0.4927	3.7003	7.0436
Fiscal expenditure decentralization	87,630	0.8314	0.0308	0.7520	0.9610
Fiscal autonomy	87,630	0.4575	0.1630	0.0980	0.9310

**Table 2:** Basis regression and endogeneity test results.

Variables	Explained Variable: Catastrophic Health Expenditure			
	(1)	(2)	(3)	(4)
Local fiscal matching subsidies	−0.0204 *** (0.0038)	−0.0143 ** (0.0064)	−1.4124 *** (0.1762)	−1.8009 *** (0.2790)
NCMS transfer payments		−0.0144 *** (0.0055)		−1.8317 *** (0.2901)
Interaction term		0.0130 *** (0.0047)		1.6090 *** (0.2602)
Age	0.0017 *** (0.0001)	0.0017 *** (0.0001)	0.0102 *** (0.0004)	0.0105 *** (0.0004)
Gender	−0.0010 (0.0021)	−0.0010 (0.0021)	−0.0047 (0.0116)	−0.0026 (0.0116)
Marital status	0.0105 *** (0.0025)	0.0103 *** (0.0025)	−0.0045 (0.0162)	0.0600 *** (0.0133)
Hukou status	0.0054 (0.0045)	0.0053 (0.0045)	0.0607 ** (0.0242)	0.0028 (0.0243)
Family size	−0.0074 *** (0.0007)	−0.0074 *** (0.0007)	−0.0367 *** (0.0036)	−0.0371 *** (0.0036)
Household health status	−0.0023 ** (0.0011)	−0.0025 ** (0.0011)	−0.0764 *** (0.0109)	0.0085 (0.0070)
Household education level	−0.0123 *** (0.0007)	−0.0124 *** (0.0007)	−0.0728 *** (0.0040)	−0.0657 *** (0.0042)
Social medical insurance	0.0594 *** (0.0055)	0.0598 *** (0.0055)	0.4822 *** (0.0348)	0.2737 *** (0.0319)
Commercial health insurance	−0.0243 (0.0203)	−0.0241 (0.0202)	−0.1210 (0.1097)	−0.0747 (0.1103)
Fiscal revenue decentralization	−0.1678 *** (0.0230)	−0.2097 *** (0.0252)	−3.3295 *** (0.3388)	−0.2609 (0.1993)
Beds	−0.0061 *** (0.0020)	−0.0042 * (0.0021)	0.0459 *** (0.0152)	−0.1505 *** (0.0304)
GDP	0.0335 *** (0.0067)	0.0304 *** (0.0067)	1.2500 *** (0.1451)	0.1859 *** (0.0339)
Hospitalization	−0.6815 *** (0.0647)	−0.5737 *** (0.0697)	1.2850 * (0.7244)	−1.1376 ** (0.4566)
Aging	0.0921 (0.0959)	0.0200 (0.0995)	−0.0878 (0.4973)	−6.4745 *** (1.1548)
F Value			73.74	99.00
Region fixed effect	Yes	Yes	Yes	Yes
Time fixed effect	Yes	Yes	Yes	Yes
Observations	87,630	87,630	87,630	87,630

Standard errors in parentheses. * *p* < 0.1, ** *p* < 0.05, *** *p* < 0.01. The coefficients are average marginal effects (Same as below).

**Table 3:** Robustness test 1.

Variables	Alternative Thresholds		Alternative Denominators	
	(1)	(2)	(1)	(2)
Local fiscal matching subsidies	−0.0366 *** (0.0088)	−0.0348 *** (0.0054)	−0.0448 *** (0.0049)	−0.0299 *** (0.0073)
NCMS transfer payments	−0.0323 *** (0.0076)	−0.0287 *** (0.0046)	−0.0440 *** (0.0041)	−0.0152 ** (0.0063)
Interaction term	0.0248 *** (0.0048)	0.0398 *** (0.0039)	0.0258 *** (0.0035)	0.0121 ** (0.0055)
Control variables	Controlled	Controlled	Controlled	Controlled
Region fixed effect	Yes	Yes	Yes	Yes
Time fixed effect	Yes	Yes	Yes	Yes
Observations	87,630	87,630	87,630	87,630

** *p* < 0.05, *** *p* < 0.01.

**Table 4:** Robustness test 2.

Variables	(1)	(2)
Local fiscal matching subsidies	−0.0117 * (0.0064)	−0.0144 ** (0.0064)
NCMS transfer payments	−0.0216 ** (0.0055)	−0.0186 *** (0.0056)
Interaction term	0.0118 ** (0.0047)	0.0142 *** (0.0047)
Fiscal expenditure decentralization	−0.4018 *** (0.0460)	
Fiscal autonomy		−0.0542 *** (0.0182)
Control variables	Controlled	Controlled
Region fixed effect	Yes	Yes
Time fixed effect	Yes	Yes
Observations	87,630	87,630

* *p* < 0.1, ** *p* < 0.05, *** *p* < 0.01.

**Table 5:** Robustness test 3.

Variables	Integration of the Urban and Rural Medical Insurance Systems		Healthcare System Reform	
	(1)	(2)	(1)	(2)
Local fiscal matching subsidies	−0.0265 *** (0.0040)	−0.0190 *** (0.0065)	−0.0236 *** (0.0039)	−0.0150 ** (0.0064)
NCMS transfer payments		−0.00137 ** (0.0056)		−0.0114 ** (0.0055)
Interaction term		0.0120 ** (0.0047)		0.0138 *** (0.0047)
Time × Urban-Rural Integration	−0.0217 *** (0.0072)	−0.0147 * (0.0075)		
Time × Healthcare Fiscal System Reform			−0.0313 *** (0.0041)	−0.0304 *** (0.0041)
Control variables	Controlled	Controlled	Controlled	Controlled
Region fixed effect	Yes	Yes	Yes	Yes
Time fixed effect	Yes	Yes	Yes	Yes
Observations	87,630	87,630	87,630	87,630

* *p* < 0.1, ** *p* < 0.05, *** *p* < 0.01.

**Table 6:** Robustness test 4.

Variables	(1)	(2)	(3)	(4)
Local fiscal matching subsidies	−0.0281 *** (0.0053)	−0.0584 *** (0.0086)	−1.5058 ** (0.7138)	−1.4255 ** (0.6573)
NCMS transfer payments		−0.0193 *** (0.0074)		−1.1579 * (0.6109)
Interaction term		0.0228 *** (0.0058)		1.3485 *** (0.5124)
Control variables	Controlled	Controlled	Controlled	Controlled
Region fixed effect	Yes	Yes	Yes	Yes
Time fixed effect	Yes	Yes	Yes	Yes
Observations	87,630	87,630	87,630	87,630

* *p* < 0.1, ** *p* < 0.05, *** *p* < 0.01.

**Table 7:** Robustness test 5.

Variables	LPM				Logit	
	(1)	(2)	(3)	(4)	(1)	(2)
Local fiscal matching subsidies	−0.0375 *** (0.0021)	−0.0260 *** (0.0040)	−0.0457 *** (0.0046)	−0.0211 *** (0.0063)	−0.2261 *** (0.0430)	−0.1549 ** (0.0717)
NCMS transfer payments			−0.0299 *** (0.0049)	−0.0161 *** (0.0054)		−0.1301 ** (0.0617)
Interaction term			0.0199 *** (0.0043)	0.0126 *** (0.0046)		0.1330 ** (0.0530)
Control variables		Controlled		Controlled	Controlled	Controlled
Constant term	0.2742 *** (0.0089)	−0.0419 (0.0534)	0.2680 *** (0.0090)	−0.0059 (0.0089)	−4.2340 (0.5994)	
Region fixed effect	Yes	Yes	Yes	Yes	Yes	Yes
Time fixed effect	Yes	Yes	Yes	Yes	Yes	Yes
R^2^	0.0045	0.0501	0.0059	0.0504		
δ	6.8595		5.0797			
E-Value					1.8175	1.7301
Observations	87,630	87,630	87,630	87,630	87,630	87,630

The results are regression coefficients. ** *p* < 0.05, *** *p* < 0.01.

**Table 8:** Robustness test 6.

Variables	(1)		(2)	
	Lower Bound	Upper Bound	Lower Bound	Upper Bound
Local fiscal matching subsidies	−1.6020	−0.0663	−3.1280	−0.1420
NCMS transfer payments			−2.4760	−0.4820
Interaction term			0.9480	2.8280

The results are UCI.

**Table 9:** Balance check.

Variables	Included Sample (N = 87,630)	Excluded Sample (N = 41,304)	Mean Difference	*p*-Value
Catastrophic health expenditure	0.1174	0.1103	0.0071	0.451
Local fiscal matching subsidies	4.1685	4.1160	0.0525	0.124
NCMS transfer payments	4.1187	4.0101	0.1086	0.271
Interaction term	8.1987	8.1260	0.0727	0.199
Age	52.3696	51.9392	0.4304	0.337
Gender	0.5010	0.5109	−0.0099	0.109
Hukou status	0.9320	0.9450	−0.013	0.029
Family size	4.6048	4.8173	−0.2125	0.018
Fiscal revenue decentralization	0.4402	0.4355	0.0047	0.176
Beds	4.8619	4.7998	0.0621	0.209
GDP	10.4745	9.9871	0.4874	0.411
Hospitalization	0.1445	0.1479	−0.0034	0.177
Aging	0.1008	0.1124	−0.0116	0.130

**Table 10:** Robustness test 7.

Variables	(1)	(2)
Local fiscal matching subsidies	−0.0700 *** (0.0212)	−0.0462 ** (0.0230)
NCMS transfer payments		−0.0701 *** (0.0219)
Interaction term		0.0169 *** (0.0063)
Control variables	Controlled	Controlled
Constant term	−0.1853 (0.3276)	−0.5998 * (0.3345)
R^2^	0.0161	0.0172
Observations	87,630	87,630

The results are regression coefficients. * *p* < 0.1, ** *p* < 0.05, *** *p* < 0.01.

**Table 11:** Results of heterogeneity test.

Variables	Geographic Location		Secondary Industry Development		Fiscal Self-Sufficiency Rate	
	Eastern	Central/ Western	Developed	Less Developed	High	Low
Local fiscal matching subsidies	−0.1551 *** (0.0088)	−0.0441 *** (0.0069)	−0.0694 *** (0.0153)	−0.0380 ** (0.0111)	−0.3782 ** (0.1112)	−0.0171 ** (0.0066)
NCMS transfer payments	−0.1486 *** (0.0090)	−0.0133 * (0.0060)	−0.0270 (0.0160)	−0.0331 *** (0.0083)	−0.2483 *** (0.1065)	−0.0196 (0.0108)
Interaction term	0.1152 *** (0.0080)	0.0252 *** (0.0051)	0.0590 *** (0.0145)	0.0131 * (0.0066)	0.2197 * (0.1044)	0.0196 *** (0.0056)
Control variables	Controlled	Controlled	Controlled	Controlled	Controlled	Controlled
Region fixed effect	Yes	Yes	Yes	Yes	Yes	Yes
Time fixed effect	Yes	Yes	Yes	Yes	Yes	Yes
Observations	25,369	62,261	53,168	34,462	23,283	64,347

The results are adjusted using the Bonferroni correction. * *p* < 0.1, ** *p* < 0.05, *** *p* < 0.01.

**Table 12:** Mediation mechanism tests.

Variables	(1)	(2)	(3)
NCMS compensation spending			0.0057 ** (0.0024)
Local fiscal matching subsidies	−0.0143 ** (0.0064)	0.5230 *** (0.0081)	−0.0130 ** (0.0064)
NCMS transfer payments	−0.0144 *** (0.0055)	−0.1077 *** (0.0072)	−0.0155 *** (0.0055)
Interaction term	0.0130 *** (0.0047)	−0.0340 *** (0.0061)	0.0121 ** (0.0047)
Control variables	Controlled	Controlled	Controlled
Region fixed effect	Yes	Yes	Yes
Time fixed effect	Yes	Yes	Yes
Observations	87,630	87,630	87,630

The second column of results are regression coefficients. ** *p* < 0.05, *** *p* < 0.01.

## Data Availability

The original data is public secondary data and can be accessed through the China Family Panel Studies (CFPS) at the following link with the permission of the Institute of Social Science Survey at Peking University: https://cfpsdata.pku.edu.cn/#/resource-detail/4 (accessed on 18 January 2026).
